# Chronic Kidney Disease in Urban, Adult Sri Lankans: A Cohort Follow‐Up Study

**DOI:** 10.1155/ijne/3236349

**Published:** 2026-05-14

**Authors:** Shamila De Silva, Dileepa Ediriweera, Madunil Niriella, Anuradhini Kasturiratne, Arunasalam Pathmeswaran, Norihiro Kato, Rajitha Wickramasinghe, H. Janaka de Silva

**Affiliations:** ^1^ Faculty of Medicine, University of Kelaniya, Colombo, Sri Lanka, kln.ac.lk; ^2^ University Medical Unit, Colombo North Teaching Hospital, Ragama, Sri Lanka, health.gov.lk; ^3^ National Center for Global Health and Medicine, Toyama Shinjuku-ku, Tokyo, Japan, ncgm.go.jp

**Keywords:** adult, chronic kidney disease, diabetes, hypertension, Sri Lanka, urban

## Abstract

**Background:**

Chronic kidney disease (CKD) affects approximately 850 million adults worldwide, with its prevalence rising due to increasing rates of diabetes and hypertension in aging populations. This study aims to assess the prevalence and associated factors of CKD in an urban adult cohort in Sri Lanka, where such data are limited.

**Methods:**

We conducted a stratified random sampling of participants from Ragama, Sri Lanka, initially in 2007 (ages 35–64) and reassessed them in 2014. Data collection involved structured interviews, anthropometric measurements, and biochemical tests. CKD was evaluated in 2014 using the estimated glomerular filtration rate (eGFR) calculated via the CKD‐EPI formula, defining CKD as eGFR < 60 mL/min/1.72 m^2^ according to KDIGO/KDOQI guidelines. A follow‐up in 2017 assessed all‐cause and cardiovascular mortality and morbidity.

**Results:**

Of 2985 individuals recruited in 2007, 2148 (71.6%) participated in the 2014 follow‐up; 2032 (94.6%) had complete data to assess CKD status in 2014. Mean age of the participants was 52.3 (SD 7.7) years; 57% were women. Age‐adjusted prevalence of CKD was 3.03 (1.98–4.11) per 100 population in 2014; most were Stage 3A (67.2%), followed by Stage 3B (23%) and Stage 4 (9.8%). Older age (*p* < 0.001), male gender (*p* < 0.05), diabetes (*p* < 0.001), and hypertension (*p* < 0.001) at baseline were associated with CKD in 2014. No association was observed between CKD and all‐cause or cardiovascular mortality or morbidity by 2017 (*p* > 0.05).

**Conclusions:**

Diabetes and hypertension are significant contributors to CKD prevalence, with most patients at Stage 3, where early detection and management can slow disease progression. CKD remains underrecognized in Sri Lanka, highlighting the need for extensive population‐based studies to better understand this growing health issue better.

## 1. Background

It is estimated that 850 million people worldwide have some degree of chronic kidney disease (CKD) [1]. Worldwide, the incidence and prevalence of CKD have doubled over the last decade and are now thought to be reaching epidemic proportions, with a population prevalence of over > 10% in many countries [1]. Despite CKD’s substantial impact on global health, it remains a low priority in public health strategies addressing the rising burden of noncommunicable diseases (NCDs) in low and middle‐income countries [2].

A systematic review in 2008 estimated the median global prevalence of CKD to be 7.2% in persons aged ≥ 30 years, increasing up to 23.4%–35.8% in persons aged ≥ 64 years [3]. A wide array of complex and closely interrelated factors contributes to the development and progression of CKD, including age, gender, genetic predisposition, obesity, hypertension, diabetes, proteinuria, dyslipidemia, and smoking [4]. CKD increases morbidity and mortality of the affected by progressing to end‐stage kidney disease (ESKD). CKD is also an independent risk factor for ischemic heart disease (IHD). Patients with CKD are at a higher risk of death due to IHD than due to CKD itself [5].

Approximately 20% of individuals with diabetes mellitus exhibit signs of diabetic nephropathy within 20 years of their diagnosis, making diabetes a primary cause of the onset and progression of CKD in many developing and developed countries and leading to significant renal complications, morbidity, diminished quality of life, and higher healthcare expenses [6–8]. Studies have also established a distinct link between nonalcoholic fatty liver disease (NAFLD) and CKD, indicating that NAFLD is independently associated with a higher prevalence of CKD [9, 10].

CKD in Sri Lanka’s North Central Province stands out globally due to a unique form of the disease, chronic kidney disease of uncertain etiology (CKDu), which disproportionately affects the farming community. Reports on the prevalence of CKDu vary significantly [11–13]. The World Health Organization (WHO) estimated a 15.3% prevalence among adults in the affected regions in 2012 [11]. However, other studies have indicated lower prevalence rates, ranging from 2% to 8.57% [12, 13]. The differing methods used to define CKDu may partially explain these discrepancies. Research on renal disease in Sri Lanka has primarily concentrated on CKDu, leading to a lack of data on the incidence and prevalence of CKD in other regions in the country.

However, it is well established that both rural and urban populations are experiencing increases in diabetes and hypertension in Sri Lanka, reflecting the global trend [14–16]. The estimated prevalence of diabetes among Sri Lankan adults was 10.3%, rising to 18.6% in the Western Province in 2011 [14]. In 2021, a systematic review and meta‐analysis found that the prevalence of diabetes and prediabetes in Sri Lanka was 12.07% and 15.57%, respectively [15]. A systematic review in 2024 estimated that the prevalence of hypertension steadily increased from 23.7% (2005–2005) to 34.8% (2021) among adults in Sri Lanka [16].

There is a lack of data on the overall community prevalence of CKD in Sri Lanka. Most published research has focused on prevalence rates within hospitals rather than examining CKD prevalence in the community [12, 17, 18]. In a resource‐limited country like Sri Lanka, preventing CKD at an early stage is crucial from a public health perspective, as managing ESKD places a significant strain on the already limited healthcare budget. To implement effective preventive strategies, gathering more information on the incidence and prevalence of CKD in regions outside the CKDu‐affected areas is essential. Identifying high‐risk groups would allow for implementing CKD screening methods using cost‐effective urine and blood tests.

The objectives of our study were to identify the prevalence and associations of CKD in a population from an urban area of the Western Province of Sri Lanka. After a 10‐year follow‐up period, the contribution of CKD to all‐cause and cardiovascular mortality in the study cohort was also assessed.

## 2. Methods

Ragama is an urban township situated 18 km north of Colombo, the commercial capital of Sri Lanka. The study population was selected from the Ragama Medical Officer of Health (MOH) area in 2007 using a cluster randomized sampling method. Each MOH area is divided into Grama Niladhari (GN) divisions, the smallest administrative units in the country. The householders’ list of the 22 GN divisions of the Ragama MOH area was used as the sampling frame. Those aged 35–64 years were stratified into three age groups of 35–44, 45–54, and 55–64 years, and a random sample was obtained from each stratum using a random number generator (PEPI statistical program). The selected participants were visited at home, and the purpose of the study, what procedures will be carried out, and the potential hazards and benefits of participation were explained, before obtaining informed written consent. This study sample is broadly representative of a Sri Lankan urban population.

Participants were screened using an interviewer‐administered structured questionnaire in 2007 and invited back after 7 years of follow‐up for reevaluation in 2014. Anthropometric indices, liver ultrasonography, and biochemical and serological tests were done on both occasions. Those with any abnormal anthropometric and metabolic derangements were referred for suitable medical care. Details of the screening process of the inception cohort in 2007 and reevaluation in 2014 are described in detail elsewhere [19, 20].

Serum creatinine was tested in 2014 using the standard Jaffe method at the same laboratory. Creatinine clearance was calculated using the CKD‐EPI formula [21]. CKD was defined as estimated glomerular filtration rate (eGFR) < 60 mL/min/1.73 m^2^, using the KDIGO/KDOQI classifications [22]. The estimated creatinine clearance (eGFR) was used to identify patients with any stage of CKD [22].

NAFLD was diagnosed on established ultrasound criteria, safe alcohol consumption (Asian standards: < 14 units/week for men, < 7 units/week for females), and absence of hepatitis B and C markers [23]. Metabolic dysfunction–associated fatty liver disease (MAFLD) was defined using standard criteria of hepatic steatosis, type 2 diabetes, overweight/obesity, and metabolic abnormalities [24].

The following exposure variables in 2007 were analyzed for association with CKD: sex, age, educational level (i.e., less than General Certificate of Education‐Ordinary Level), income (i.e., less than the median income in the cohort), unsafe alcohol use (Asian cutoff of > 14 units/week for men and > 7 units/week for women), smoking pack‐years, prediabetes (HbA1c :5.7%–6.4%), central obesity (waist circumference ≥ 90 cm for males and ≥ 80 cm for females), raised blood pressure (systolic BP ≥ 140 or diastolic BP ≥ 90 mmHg, or treatment of previously diagnosed hypertension), low HDL (< 40 mg/dL [1.03 mmol/dL] in males and < 50 mg/dL [1.29 mmol/dL] in females, or on specific treatment for this lipid abnormality), raised triglycerides (≥ 150 mg/dL [1.7 mmol/L] or on specific treatment for this lipid abnormality), dyslipidemia (total cholesterol > 140 mg/dL, raised TG, raised HDL, or on lipid‐lowering therapy), and NAFLD.

At the third evaluation in 2017 (10 years after recruitment), participants were assessed for fatal and nonfatal cardiovascular events (CVEs), and the identified CVEs were confirmed by examination of the relevant medical records/death certificates. Nonresponders were contacted by telephone or by post. With informed consent, the participant (or a collateral historian, in case the participant was deceased) was interviewed, and details regarding whether the participant had suffered any fatal or nonfatal CVEs during the previous 10 years were obtained. These CVEs were confirmed by medically trained research assistants, based on examination of death certificates and hospital records [25].

Nonfatal CVEs were identified either by documentation in medical records or, in the absence of medical records, by the presence of an episode with clinically significant chest pain or clinically significant neurological deficit, or intervention for either. Clinically significant chest pain was defined as chest pain requiring hospital admission for more than 24 h and requiring special treatment (streptokinase, heparin, cardiology referral with or without echocardiography, and/or referral for coronary intervention). A clinically significant neurologically deficit was defined as a sudden onset neurological deficit lasting more than 24 h, requiring a CT scan of the brain and/or management in a specialized stroke unit.

Endpoints assessed were fatal CVE (death from myocardial infarction or stroke) and nonfatal CVE (nonfatal myocardial event [NFME], nonfatal stroke [NFS], coronary artery bypass surgery [CABS], and percutaneous transluminal coronary angiography [PTCA]).

To identify baseline predictors of CKD in 2017, a parsimonious multiple logistic regression model was constructed using forward selection. This iterative process began with a null model (intercept only), and at each step, the variable that most significantly improved model fit (likelihood ratio test, *p* < 0.05) was added. The forward selection continued until no remaining variable met the inclusion criterion (*p* ≥ 0.05).

The association between CKD and all‐cause and cardiovascular mortality and morbidity in 2017 was assessed using right‐censored survival data. Follow‐up time was defined as the interval between the 2007 baseline assessment and the date of the outcome (death or cardiovascular morbidity) or censoring (last confirmed alive). Univariable Cox proportional hazard models were fitted for each outcome, with CKD status as the predictor. Hazard ratios (HRs) were calculated for each outcome. Statistical significance was defined as *p* < 0.05. All analyses were performed using R programming language Version 4.4.2 (2024‐10‐31 ucrt).

Ethical approval for all phases of the study was obtained from the Ethics Review Committee of the Faculty of Medicine, University of Kelaniya (P38/09/2006, P/169/08/2014, and P/133/05/2017).

## 3. Results

Of 2985 individuals recruited in 2007, 2148 (71.6%) attended follow‐up in 2014. 2032/2148 (94.6%) had data on CKD (57.0% women, mean age: 52.3 [SD: 7.7] years).

61/2032 (3%) participants met standard CKD diagnostic criteria of eGFR < 60 mL/min/1.73 m^2^. Age‐adjusted prevalence of CKD was 3.03 (1.98–4.11) per 100 population in 2014 (Table 1). For every 5‐year increment in age, the odds ratio for developing CKD increased by 1.80.

**TABLE 1:** Age‐adjusted prevalence of CKD in 2014.

Age stratum (years)	Prevalence per 100	95% confidence interval
35–44	0.29	0.00–0.84
45–54	1.64	0.75–2.53
55–65	5.27	3.81–6.74

The distribution of CKD by stage and albuminuria is shown in Tables 2 and 3. The majority of those with CKD were in Stage 3A.

**TABLE 2:** Distribution of CKD by stage.

CKD stage	Number of participants (%)
1	1530 (75.3)
2	441 (21.7)
3A	41 (2)
3B	14 (0.7)
4	6 (0.3)
5	0

**TABLE 3:** Distribution of CKD by stage and albuminuria.

CKD grade	uACR (mg/g)		
eGFR (mL/min/1.73 m^2^)	< 0.2	0.2–200	> 200
G 1 (≥ 90)	1483	37	0
G 2 (60–89)	427	12	0
G 3a (45–59)	36	5	0
G 3b (30–44)	11	3	0
G 4 (15–29)	4	2	0
G 5 (< 15)	0	0	0

Compared to those with eGFR > 60 mL/min/1.72 m^2^ in 2014, those with CKD were likely to be male, older, have diabetes, hypertension, or increased serum triglycerides (or be on treatment for this abnormality) in 2014 (Table 4).

**TABLE 4:** Characteristics in 2014 of those with and without established CKD in 2014.

Parameter in 2014	CKD (eGFR ≤ 60) *n* = 61	Non‐CKD (eGFR > 60) *n* = 1971	*p* value
Male gender	36 (59.0%)	837 (42.5%)	0.015
Age (years)	66.0 (61.0–68.0)	60.0 (54.0–65.0)	< 0.001
Low education level (less than GCE O/L)	26 (42.6%)	860 (43.8%)	0.958
Household income < median of cohort	29 (49.1%)	768 (39.8%)	0.189
Weight (kg)	61.3 (53.1–68.1)	59.6 (52.3–68.1)	0.393
Waist (cm)	87.0 (77.8–92.8)	85.1 (78.2–92.0)	0.434
BMI (kg/m^2^)	25.0 (21.6–26.7)	24.3 (21.7–27.2)	0.942
BMI > 23 kg/m^2^	40 (65.6%)	1268 (64.4%)	0.958
Central obesity	34 (56.7%)	1088 (55.4%)	0.953
Diabetes	41 (67.2%)	795 (40.4%)	< 0.001
Prediabetes	14 (70.0%)	839 (71.8%)	1.000
Hypertension	48 (78.7%)	982 (49.8%)	< 0.001
Elevated TG or on treatment	42 (70.0%)	918 (46.6%)	< 0.001
Low HDL or on treatment	36 (60.0%)	1002 (50.9%)	0.206
Dyslipidemia	50 (82.0%)	1375 (70.0%)	0.057
NAFLD	39 (63.9%)	1249 (63.4%)	1.000
MAFLD	39 (63.9%)	1249 (63.4%)	1.000
Coronary artery disease	5 (8.2%)	107 (5.4%)	0.518

*Note:* MAFLD, metabolic dysfunction–associated fatty liver disease; GCE O/L, General Certificate of Education Ordinary Level examination.

Abbreviations: BMI, body mass index; NAFLD, nonalcoholic fatty liver disease.

Those who had developed CKD by 2014 were likely to be older, male, with diabetes and hypertension, have high serum triglycerides or be on treatment for this abnormality, and have MAFLD in 2007 (Table 5).

**TABLE 5:** Characteristics in 2007 of those with and without established CKD in 2014.

Parameter in 2007	CKD in 2014 *n* = 61	No CKD in 2014 *n* = 1971	*p* value
Age (years)	60.0 (55.0–62.0)	53.0 (47.0–58.0)	< 0.001
Male gender	36 (59.0%)	837 (42.5%)	0.015
Low education level (less than GCE O/L)	26 (42.6%)	860 (43.8%)	0.958
Household income < median of cohort	28 (45.0%)	846 (43.8%)	0.841
Smoking > 1 pack year	8 (13.1%)	248 (12.6%)	1.000
Unsafe alcohol	9 (14.7%)	343 (17.4%)	0.714
Weight (kg)	61.7 (54.0–69.8)	59.2 (52.3–67.2)	0.263
Waist (cm)	87.5 (80.1–94.3)	85.9 (79.2–92.6)	0.555
BMI (kg/m^2^)	24.2 (21.3–26.6)	24.1 (21.4–26.8)	0.879
BMI > 23 kg/m^2^	39 (63.9%)	1210 (61.6%)	0.811
Central obesity	33 (54.1%)	1115 (56.6%)	0.801
Diabetes	27 (44.3%)	340 (17.3%)	< 0.001
Prediabetes	14 (41.2%)	409 (25.1%)	0.053
Hypertension	44 (72.1%)	823 (41.8%)	< 0.001
Elevated TG or on treatment	33 (54.1%)	692 (35.1%)	0.003
Low HDL or on treatment	19 (31.1%)	533 (27.0%)	0.573
Dyslipidemia	44 (72.1%)	1254 (63.6%)	0.219
NAFLD	27 (44.3%)	641 (32.5%)	0.142
MAFLD	30 (49.2%)	665 (33.8%)	0.019
Coronary artery disease	2 (3.2%)	76 (3.9%)	1.000

*Note:* MAFLD, metabolic dysfunction–associated fatty liver disease; GCE O/L, General Certificate of Education Ordinary Level examination.

Abbreviations: BMI, body mass index; NAFLD, nonalcoholic fatty liver disease.

Independent predictors of CKD, identified through multiple logistic regression, were older age (*p* < 0.001), male gender (*p* = 0.004), and presence of diabetes (*p* < 0.001) and hypertension (*p* = 0.004) in 2007 (Table 6). Specifically, every 5‐year increase in age was associated with an 80% increase in the odds of having CKD (OR: 1.80, 95% CI: 1.42–2.33). Being male more than doubled the odds (OR: 2.19, 95% CI: 1.29–3.77). Having diabetes tripled the odds (OR: 3.11, 95% CI: 1.81–5.28), and having hypertension was associated with 2.37 times higher odds (OR: 2.37, 95% CI: 1.34–4.37).

**TABLE 6:** Characteristics of 2007 that are associated with CKD on multivariate analysis (independent predictors of CKD).

Variable	OR (95% CI)	*p* value
Age (every 5 years)	1.80 (1.42–2.33)	< 0.001
Male sex	2.19 (1.29–3.77)	0.004
Diabetes	3.11 (1.81–5.28)	< 0.001
Hypertension	2.37 (1.34–4.37)	0.004

Survival analysis results are shown in Table 7. For all‐cause mortality, the HR was 2.069 (0.6442–6.642, *p* = 0.222), indicating no association. For cardiovascular disease (CVD) mortality, the HR was 1.429 (0.193–10.58, *p* = 0.727), also showing no association. Similarly, for CVD morbidity, the HR was 1.829 (0.8059–4.15, *p* = 0.149), which was not significant. Therefore, CKD in 2014 was not associated with all‐cause mortality, CV mortality, or morbidity (*p* > 0.05) in 2017.

**TABLE 7:** CKD as an independent risk factor for all‐cause and CV mortality and morbidity.

	**Hazard ratio**	***p* value**

All‐cause 2017	2.069 (0.6442–6.642)	0.222
CVD mortality 2017	1.429 (0.193–10.58)	0.727
CVD morbidity	1.829 (0.8059–4.15)	0.149

*Note:* Individual variable analysis: Cox regression.

## 4. Discussion

This study is the first community‐based cohort follow‐up epidemiological research conducted in Sri Lanka to investigate the prevalence of CKD in a region where CKDu is not prominent. The findings highlight the significant impact of CKD in Sri Lanka and emphasize the importance of reducing the future burden of CKD in a densely populated area of the country. The age‐adjusted prevalence of CKD was 3.03 per 100 population in 2014; most cases were Stage 3A. Older age, male gender, diabetes, and hypertension at baseline were found to be associated with CKD in 2014. No association was observed between CKD in 2014 and all‐cause or cardiovascular mortality or morbidity by 2017.

Previous research estimates that the prevalence of CKD due to known etiology in Sri Lanka is 0.4 per 1000 individuals [26]. The prevalence rates observed in the current study were similar. A study from the Kidney Disease Screening project reported a CKD prevalence of 17.2% among urban adults in India. This study included a mix of urban and peri‐urban populations and used the MDRD equation to estimate GFR [27]. A 2014 rural population study in Karnataka found 6.3% CKD prevalence using the MDRD equation among adults aged ≥ 18, with higher rates in older adults and those with diabetes or hypertension [27]. A community‐based study conducted in Karachi, Pakistan, reported a CKD prevalence of 12.5% among adults aged ≥ 40 years. This was based on an eGFR < 60 mL/min/1.73 m^2^ or albuminuria, using the CKD‐EPI equation [28]. A systematic review reported a pooled CKD prevalence of 17.3% among Bangladeshi adults, with individual studies showing rates ranging from 12.8% to 26% [29]. No CKD prevalence data are available for many other South Asian countries. Methodological variations (e.g., MDRD vs. Cockcroft–Gault equations) affect comparability.

Older age is a well‐known risk factor for the development of CKD [30]. For every 5‐year increment in age, the odds ratio for developing CKD increased by 1.80 in the present study, with the prevalence of CKD in the 55–65 age group being 5.27 per 100 population.

Fifty‐nine percent of persons with CKD in the study cohort were male. This finding aligns with previous research indicating that men are at a higher risk of developing CKD [31, 32]. However, some studies have reported a higher prevalence of CKD among women [33–35]. This discrepancy suggests that gender‐related risk factors for CKD may vary across different populations and highlights the need for early detection in both men and women. Further research is needed to understand the underlying mechanisms.

Interestingly, the distribution of CKD stages in the study cohort followed the well‐known iceberg shape of CKD prevalence recognized in most populations. It is thought that for each patient with ESKD, there are more than 100 with various stages of CKD in the community [36]. There were no subjects in Stage 5 (ESKD) in our sample, and only 6 (0.3%) were in Stage 4. The majority (41%–2%) were in Stage 3A where early recognition and better control of comorbid factors are known to retard progression. There were 1530 (75.3%) and 441 (21.7%) persons in CKD Stages 1 and 2, respectively. These early stages of CKD comprised 97% of this older aged cohort. Although they do not fall within the standard definition of CKD (eGFR < 60 mL/min/1.73 m^2^), it is important to detect these early stages of CKD in the community, since these persons need regular follow‐up.

CKD was not associated with all‐cause mortality, CV mortality, or morbidity in 2017 in the study participants. This is probably attributable to the short follow‐up period of only 3 years. We used a single serum creatinine value to identify CKD as per standard worldwide research practice since subjects were from the community with most probably stable serum creatinine for 3 months or more [37]. Formulas used to calculate eGFR are known to systematically underestimate GFR. CKD‐EPI used in this research performs better at higher GFR values than the MDRD equation, but performance in the elderly remains uncertain [21, 22].

The key strengths of this study are its large sample size, 7‐year follow‐up period, and reasonable follow‐up attendance. To the best of our knowledge, this is the first community‐based study looking at CKD in the areas outside the CKDu belt in Sri Lanka. The study addresses a knowledge gap since it explicitly targets the lack of community‐based CKD prevalence data in Sri Lanka outside of CKDu areas, helping to understand the broader CKD burden in the country. The longitudinal cohort design allows for the assessment of risk factors at baseline and the subsequent development of CKD, providing stronger evidence for associations than cross‐sectional studies.

There are some limitations in this research. CKD was determined using a single eGFR measurement, which may have a misclassification bias. Serum creatinine was not measured in 2007. The study only analyzed data from a single province in Sri Lanka, which limits the generalizability of its findings. Although ultrasound data on NAFLD were available, this research did not specifically look for features of CKD on ultrasound scans.

## 5. Conclusions

In this community‐based epidemiological study looking at prevalence rates of CKD in an area outside the CKDu belt, the prevalence of CKD among older adults in urban Sri Lanka appears to be quite high. The findings underscore the importance of screening for CKD, at least in the at‐risk populations, in parts of the country where CKDu is not widely prevalent. CKD appears to be underrecognized in Sri Lanka, and large population‐based studies are needed to better understand the size of the problem so that preventive strategies can be implemented more effectively.

**Nomenclature**
ACR: Albumin to creatinine ratio
CABS: Coronary artery bypass surgery
CKD: Chronic kidney disease
CKD‐EPI: Chronic Kidney Disease Epidemiology Collaboration
CKDu: Chronic kidney disease of uncertain etiology
CV: Cardiovascular
CVD: Cardiovascular disease
CVEs: Cardiovascular events
eGFR: Estimated glomerular filtration rate
ESKD: End‐stage kidney disease
GN: Grama Niladhari
HR: Hazard ratio
IHD: Ischemic heart disease
KDIGO/KDOQI: Kidney Disease Improving Global Outcomes/Kidney Disease Outcomes Quality Initiative
MAFLD: Metabolic dysfunction–associated fatty liver disease
MDRD: Modified diet in renal disease
MOH: Medical Officer of Health
NAFLD: Nonalcoholic fatty liver disease
NCDs: Noncommunicable diseases
NFME: Nonfatal myocardial event
NFS: Nonfatal stroke
PTCA: Percutaneous transluminal coronary angiography
RHS: Ragama Health Study
WHO: World Health Organization

## Author Contributions

Shamila De Silva and H. Janaka de Silva conceptualized and designed the study. Anuradhini Kasturiratne, Shamila De Silva, Madunil Niriella, Rajitha Wickramasinghe, Norihiro Kato, and H. Janaka de Silva were involved in establishing the Ragama Health Study cohort. Shamila De Silva, Madunil Niriella, and Anuradhini Kasturiratne collected data. Dileepa Ediriweera analyzed the data assisted by Shamila De Silva. Shamila De Silva, and Dileepa Ediriweera prepared and revised the manuscript.

## Funding

We gratefully acknowledge grants from the Ministry of Higher Education of Sri Lanka and the National Center for Global Health and Medicine, Tokyo, Japan, for conducting this study.

## Disclosure

A previous version of this manuscript was published as a preprint and is available at the following link: https://www.researchsquare.com/article/rs-6233683/v1. All authors read and agreed to the final version of the manuscript. The funding bodies played no role in the design of the study, collection, analysis, and interpretation of data, and in writing the manuscript.

## Ethics Statement

Ethical approval was obtained from the Ethics Review Committee of the Faculty of Medicine, University of Kelaniya. Informed written consent was obtained from all participants in 2007, 2014, and 2017 (Approval Nos. P/38/09/2006, P/169/08/2014, and P/133/05/2017).

## Conflicts of Interest

The authors declare no conflicts of interest.

## Data Availability

The datasets used and analyzed during the current study are available from the corresponding author upon reasonable request.
